# Performance of thermodilution catheters under control and extreme circulatory conditions in a pig model

**DOI:** 10.1186/cc9466

**Published:** 2011-03-11

**Authors:** XX Yang, LA Critchley, F Zhu, Q Tian

**Affiliations:** 1 The Chinese University of Hong Kong

## Introduction

When validating new methods of cardiac output, measurement comparisons are made using Bland-Altman and percentage errors are generated that rely on a precision error for thermodilution of ± 20% [1]; data collected 30 years ago [2]. We have re-evaluated this precision against an aortic flow probe.

## Methods

Four domestic pigs, weight 30 to 32 kg, were anaesthetized. An aortic flow probe was placed via a left thoracotomy. Both Arrow (*n *= 6) and Edwards (*n *= 6) 7F pulmonary artery catheters and a Siemens SC9000 monitor were used. Sets of cardiac output readings were taken (three to six pairs). Catheters were changed frequently and cardiac output increased (for example, dopamine and adrenaline) and decreased (for example, trinitrate and beta-blocker) using drug infusions. Baseline and drug treatment data were compared.

## Results

**Figure 1:**
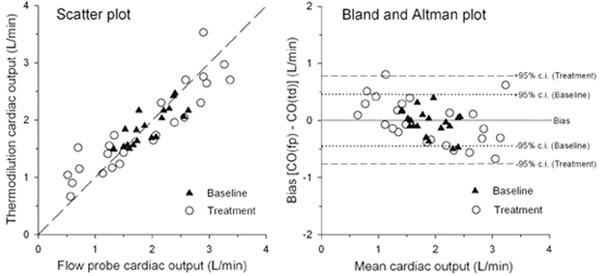
**Plots showing widening distribution**.

Forty-five sets (259 pairs) of averaged data (21 baseline and 24 following treatment) were collected. Baseline cardiac outputs (mean (SD)) were 1.9 (0.4) and 1.8 (0.3) l/minute for flow meter and thermodilution readings, respectively. MAP (mean (range)) was 82 (69 to 95) mmHg. Following circulatory treatment, cardiac output ranged from 0.5 to 3.4 l/minute and from 0.7 to 3.5 l/minute, respectively. MAP ranged from 44 to 118 mmHg. For baseline data, bias was 0.0 l/minute, limits of agreement ± 0.45 l/minute and percentage error ± 24.3%. Following treatment, the bias was unchanged at 0.0 l/minute, but the limits of agreement widened to ± 0.78 l/minute and percentage error widened to 42.0% (Figure 1).

## Conclusions

The flow probe has a relatively low (1 to 2%) precision error, thus the baseline percentage error of 24.3% is in keeping the quoted precision error for thermodilution of ± 20%. However, under more extreme circulatory conditions thermodilution behaved less reliably with widened limits of agreement and precision errors (42.0%). Thermodilution is less accurate than originally thought in haemodynamically unstable patients.
